# Revolutionizing cervical cancer treatment: single-cell sequencing of *TSPAN1+* tumor EPCs and immune checkpoints to assess drug sensitivity and optimize therapy

**DOI:** 10.3389/fimmu.2025.1574174

**Published:** 2025-07-24

**Authors:** Yumeng Li, Zhiheng Lin, Guangyao Lin, Zhijie Zhao, Zhikai Xiahou, Pingping Cai

**Affiliations:** ^1^ College of First Clinical Medicine, Shandong University of Traditional Chinese Medicine, Jinan, China; ^2^ Department of Gynecology, Longhua Hospital, Shanghai University of Traditional Chinese Medicine, Shanghai, China; ^3^ Department of Plastic and Reconstructive Surgery, Shanghai Ninth People’s Hospital, School of Medicine, Shanghai Jiao Tong University, Shanghai, China; ^4^ China Institute of Sport and Health Science, Beijing Sport University, Beijing, China; ^5^ Department of Traditional Chinese Medicine, Shandong Provincial Hospital Affiliated to Shandong First Medical University, Jinan, China

**Keywords:** cervical cancer, tumor microenvironment, endothelial progenitor cells, immune checkpoints, drug sensitivity

## Abstract

**Background:**

Cervical cancer’s tumor microenvironment (TME) was composed of a diverse array of immune cells that significantly influence tumor progression and response to treatment. Recent advancements in multi-omics and single-cell sequencing had provided valuable insights into the cellular heterogeneity and immune landscape of the TME, revealing critical interactions that shape tumor behavior and therapy outcomes.

**Method:**

This study used multi-omics and single-cell sequencing to explore the immune landscape, cellular heterogeneity, and drug sensitivity in cervical cancer, focused on tumor subtypes and their interactions with immune cells, and aimed to understand therapy responses.

**Results:**

The research presented a thorough single-cell analysis of cervical cancer, identified distinct tumor epithelial cell (EPC) subtypes, and explored their roles in tumor progression, immune evasion, and therapeutic response. It underscored the potential of tumor EPCs as valuable biomarkers for prognosis and as targets for personalized treatment approaches.

**Conclusion:**

The immune landscape of cervical cancer and its interaction with tumor endothelial progenitor cells played crucial roles in determining the tumor’s progression and response to therapy. The classification of tumor subtypes based on immune characteristics and drug sensitivity was critical for personalized treatment. The identification of *TSPAN1* as key biomarkers provided insight into tumor biology and potential therapeutic targets. Our findings emphasized the need for combining immune checkpoint modulation with precise drug sensitivity analysis to optimize treatment strategies, particularly in advanced cervical cancer.

## Introduction

As one of the most common cancers in women, cervical cancer affected the cervix. The cervix, located at the lower part of the uterus, connected the uterine body and the vagina, serving as the birth canal during childbirth (1). Cervical cancer primarily occurred at the squamocolumnar junction, where the squamous epithelium and columnar epithelium meet. Infection with high-risk HPV types, particularly 16, 18, 31, and 33, was the main cause of cervical cancer (2). Over 90% of cervical cancer patients were found to be infected with high-risk HPV. As a primary preventive tool, the HPV vaccine reduced high-risk HPV infection and cervical cancer incidence (3). Advances in cervical cancer screening, such as liquid-based cytology and HPV DNA testing, allowed for earlier detection of precancerous lesions and early intervention (4).

Traditional treatment methods for cervical cancer included surgery, radiotherapy, and chemotherapy. Surgery was suitable for early-stage cervical cancer, such as total hysterectomy or radical hysterectomy; radiotherapy and chemotherapy were used for cervical cancer at all stages, especially advanced or recurrent cervical cancer (5, 6). In recent years, new treatment strategies, such as targeted therapy, immunotherapy, and therapeutic vaccines, had emerged (7). Targeted therapies based on viral gene integration, oxidative stress, VEGF, EGFR, and PD-1 signaling were applied. Clinical trials indicated promising results for immune checkpoint inhibitors and other immunotherapies (8).

Immunotherapy for cervical cancer has made significant progress in recent years, with immune checkpoint inhibitors becoming the main focus of research. Pembrolizumab and nivolumab, PD-1/PD-L1 inhibitors, were included in the National Comprehensive Cancer Network guidelines as first-line treatments for PD-L1-positive advanced cervical cancer based on their clinical efficacy (9). Moreover, the combination of CTLA-4 inhibitor ipilimumab with PD-1 inhibitors, known as dual immunotherapy, has further enhanced immune responses, thereby increasing disease control rates and objective response rates (10, 11). In addition, adoptive T cell therapies, including TILs, TCR-T, and CAR-T cell therapies, have demonstrated enormous potential by modifying or expanding the patient’s own T cells to improve their ability to attack tumor cells. Furthermore, combination therapy strategies, such as immunotherapy combined with chemotherapy, radiotherapy, and targeted therapy, have improved efficacy, thus extending progression-free survival and overall survival (12, 13). Notably, novel immunotherapeutic agents, like the bispecific antibody cadonilimab, have also shown good clinical outcomes, especially in PD-L1-negative patients (14, 15). Looking ahead, future research will focus on exploring the application of immunotherapy in frontline cervical cancer treatment, as well as identifying biomarkers to predict efficacy (16, 17). Further research will examine the long-term safety and adverse reactions to refine personalized treatment for greater precision and effectiveness.

Single-cell sequencing technology advanced the study of cervical cancer, particularly in tumor microenvironment (TME) and tumor cell heterogeneity. Through single-cell sequencing (18), TME characteristics of cervical cancer were revealed, identifying crucial subpopulations involved in immune responses (19–21) Similarly, in studies on tumor cell heterogeneity, single-cell sequencing had helped analyze cervical cancer tissues with different HPV integration statuses, showing that tumor cells with transcriptional HPV integration exhibited reduced HLA-related antigen expression and increased expression of immune checkpoint ligands (22). This finding indicated that HPV integration status had led to transcriptional reprogramming of tumor cells, thereby enabling immune evasion (23). By identifying biomarkers associated with cervical cancer development, single-cell sequencing showed promise for clinical application. Constructing the single-cell immune landscape at different stages of cervical cancer helped us track immune changes, providing insights for targeted treatments (24). In summary, single-cell sequencing advanced cervical cancer research by uncovering its biological complexity, aiding early diagnosis, prognosis, and targeted treatment (25).

## Materials and methods

### Get cervical cancer data

Cervical cancer single-cell RNA sequencing (scRNA-seq) data were accessed from the GEO database (GSE208653), and bulk RNA-seq data, with clinical and mutation information, were retrieved from TCGA. Ethical approval was not necessary as the data were publicly available (26–28).

### Analyzing raw data through processing and visualization

R software (version 4.3.0) and Seurat (v4.1.1) (29, 30) were used to analyze the 10X genomics data. DoubletFinder (v2.0.3) identified and removed doublets (31, 32) while low-quality cells were filtered based on nFeature (300–6,000), nCount (500–75,000), mitochondrial gene expression (<25%), and red blood cell gene expression (<5%). The data were normalized, the top 2,000 highly variable genes selected. ScaleData functions prepared the gene expression data guiding subsequent principal component analysis (PCA) (33), and batch correction were applied using Harmony (v0.1.1) mitigated batch effects between sample. Seurat’s FindClusters and FindNeighbors functions are used initially to identify cell clusters. Uniform manifold approximation and projection (UMAP) (34) was used to transform high dimensional data into a lower dimensional 2D space.

### Investigation into cancer preferences

To analyze different cell types and tumor cell subtypes’ cancer preference, odds ratios (OR) were calculated using the standard method (35).

### Enrichment analysis coupled with AUCell

We performed functional analysis using Gene Ontology (GO) (36, 37), analyzing Gene Ontology Biological Process (GOBP) and Kyoto Encyclopedia of Genes and Genomes (KEGG) (38) with the ClusterProfiler R package (version 4.6.2) (39). Gene Set Enrichment Analysis (GSEA) was applied to evaluate gene set expression patterns (40). Additionally, AUCell was used to identify active gene sets in our scRNA-seq data. The AUCell R package assessed stemness gene set enrichment by ranking them using the “AUCell_buildRankings” function. Gene set variation analysis (GSVA) was conducted to evaluate gene expression variability and enrichment in each sample.

### InferCNV for tumor cell detection

CNV analysis of scRNA-seq data was performed using the inferCNV R package (version 1.6.0) (41, 42). The analysis combined gene expression and chromosomal location data to assess the CNV status of chromosomes in each cell, enabling efficient distinction between malignant and normal cells.

### Identification and annotation of cell types

We begin by extracting cells for each major cell type from the integrated overview dataset. Next, these major cell types undergo integration for further subclustering. Following integration, we scale genes to unit variance. As a final step, we apply scaling, PCA, and clustering as outlined earlier. Cell clusters were initially identified with Seurat’s FindClusters and FindNeighbors functions (43). Clusters were annotated based on marker gene expression averages. Differentially expressed genes (DEGs) among clusters were identified using FindAllMarkers (44), tumor cells were re-clustered and classified by specific marker genes to explore their heterogeneity.

### Trajectory analysis of lineages

Tumor cell subtypes were ranked by differentiation using CytoTRACE. Slingshot (v2.6.0) was then applied to infer cell lineages, and the “getLineages” and “getCurves” functions estimated expression levels, aiding in the understanding of differentiation trajectories (45).

### Cellular interaction and signaling

Cell interactions, including those between tumor subtypes and other cells, were visualized using the CellChat R package (v1.6.1). Intercellular interactions were inferred from scRNA-seq data. The “netVisual_diffInteraction” function was used to visualize communication intensity differences, and “identifyCommunicationPatterns” helped identify communication patterns. The CellChat database (http://www.cellchat.org/) was also consulted to predict signaling pathways and interactions, with a p-value cutoff of 0.05.

### SCENIC-based reconstruction of gene regulatory networks

To reconstruct gene regulatory networks and identify stable cell states from scRNA-seq data, we applied the pySCENIC (v0.10.0) in Python (v3.7). AUCell matrices were used to evaluate the enrichment of transcription factors (TFs) and regulatory factor activity (46).

### Establishment and validation of a predictive model

Significant prognostic genes were identified using univariate Cox and Lasso regression analyses (47–49). Multivariate Cox regression was then applied to calculate risk coefficients, forming a risk score model (Risk score = $\sum_{\text{i}}^{\text{n}}\text{Xi}\times \text{Yi}$ ). Patients were classified based on the optimal cut-off value and the “surv_cutpoint” function (50). Survival analysis was performed using the Survival package in R (version 4.3.0), and curves were visualized with ggsurvplot (51). The model’s accuracy was evaluated by generating receiver operating characteristic (ROC) curves with the timeROC package (version 0.4.0). To assess the classification performance of the model, we use the area under the ROC curve (AUC) (52).

We validated the independence of the risk score with multivariate Cox regression and developed a Nomogram to predict overall survival (OS). Internal validation was performed using the C-index and calibration curves (53).

### Immune microenvironment assessment and analysis

The CIBERSORT R package (version 0.1.0) was employed to score immune cells. We used CIBERSORT, ESTIMATE, and Xcell to evaluate the immune microenvironment, including immune infiltration and checkpoint gene expression (54–56). We examined correlations between immune cells, model genes, OS, and risk scores. Tumor immunotherapy response was evaluated using the TIDE tool (http://tide.dfci.harvard.edu) (57).

### Assessment of drug sensitivity

To relate our findings to clinical drug use, we evaluated drug sensitivity with the “pRRophetic” package (version 0.5), determining half maximal inhibitory concentration (IC50) values for each sample and comparing responses between high and low-risk groups (58).

### Cell cultivation

Ca Ski and MS751 cells were cultured in RPMI-1640 medium with 10% FBS and 1% antibiotics at 37°C with 5% CO2 and 95% humidity to ensure optimal growth conditions.

### Application of cell transfection

To knock down *CDC42EP5* expression, cells were transfected with RNA constructs from GenePharma (Suzhou, China). Cells, seeded at 50% confluence in a 6-well plate, were transfected with si-CDC42EP5-1, si-CDC42EP5-2, or a control si-RNA (si-NC) using Lipofectamine 3000RNAiMAX reagent (Invitrogen, USA), as instructed by the manufacturer. Additional si-RNAs from RIbio (China) were also introduced.

### Western blot analysis

At 70% confluence, the transfected cells were lysed with RIPA buffer, and lysates were centrifuged at 12,000 rpm for 15 minutes (59). The supernatants were analyzed by SDS-PAGE and transferred to PVDF membranes. After blocking with 5% BSA for 1.5 hours, the membranes were incubated overnight with Anti-CDC42EP5 antibody at 4°C. After a 1-hour incubation with a secondary antibody, *CDC42EP5* was detected via enhanced chemiluminescence.

### qRT-PCR: gene expression quantification

RNA extraction was performed using Trizol reagent, followed by reverse transcription with the PrimeScript™ Kit. Gene expression was measured using qRT-PCR with SYBR Green (60, 61).

### Viability analysis of cells

Cell survival in Ca Ski and MS751 cells was assessed using the CCK-8 assay. Cells (5×10³/well) were incubated in 96-well plates for 24 hours, followed by the addition of 10μL CCK-8 reagent (62). Plates were incubated at 37°C for two hours, and absorbance at 450 nm was recorded daily for four days. The survival pattern was plotted using mean optical density values.

### Transwell migration and invasion experiment

Before starting the experiment, cells were serum-starved for 24 hours in serum-free medium. They were then suspended in Matrigel and placed in the upper compartment of Costar transwell plates, with serum-containing medium in the lower compartment to create a chemotactic gradient. After 48 hours, cells were fixed with 4% paraformaldehyde and stained with crystal violet to assess invasion.

### Wound healing assay: cell migration study

Stably transfected cells were cultured to confluence in 6-well plates. Uniform scratches were made with a 200μL pipette tip, and the wells were rinsed with PBS. After incubation in serum-free medium, the wound area was photographed at 0 and 48 hours. Image-J software measured the scratch width to analyze migration.

### EdU proliferation assay experiments

Ca Ski and MS751 cells were transfected and seeded in 6-well plates at 5×10³ cells per well. After 24 hours, EdU reagent was added to label DNA for 2 hours. The cells were washed, fixed, permeabilized, and stained with Apollo and Hoechst dyes. Proliferation was analyzed by fluorescence microscopy.

### Analysis of statistical data

Statistical analysis was done using R (v4.3.0) and Python (v4.2.0), with Wilcoxon’s test and Pearson’s correlation coefficient applied to evaluate differences. We defined significance as *P< 0.05, **P< 0.01, ***P< 0.001, and ****P< 0.0001, with “ns” for non-significant results. This approach ensured the robustness of our conclusions (63, 64).

## Results

### Single cell landscape of cervical cancer and its analysis of tumor EPCs subtypes were explored

**Figure 1:**
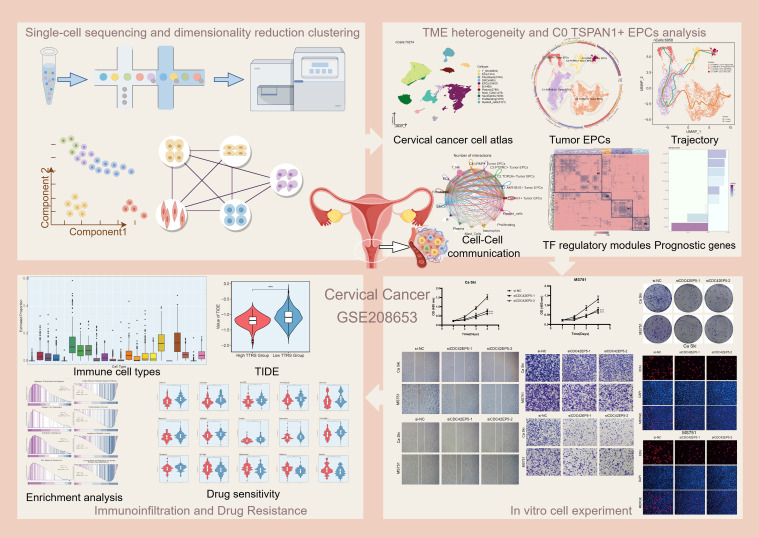
Graphical abstract. Workflow diagram for single-cell sequencing analysis of the GSE208653 dataset.

**Figure 2:**
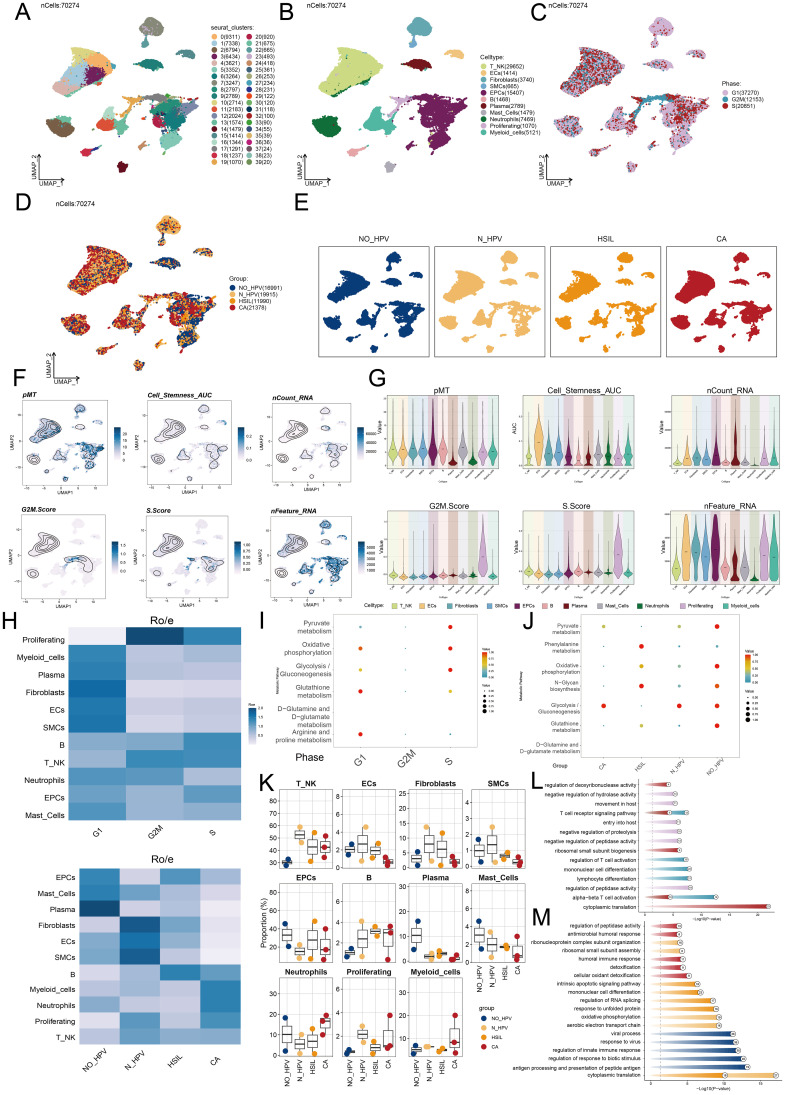
Single-cell profiling of cervical cancer. **(A)** The UMAP plot depicted the distribution of fourty seurat clusters across the entire cell population. **(B)** The UMAP plot displayed the distribution of eleven different cell types, including T cell and NK cells, ECs, Fibroblasts, SMCs, EPCs, B cells, Plasma cells, Mast cells, Neutrophils, Proliferating cells, and Myeloid cells. **(C)** The UMAP plot illustrated the distribution of different cell cycle phases. **(D, E)** The UMAP plots illustrated the distribution of four groups including NO-HPV, N-HPV, HSIL, CA. **(F)** The UMAP plots was employed to visualize the distribution of pMT, Cell Stemness AUC, nCount-RNA, G2/M.Score, S.Score, and nFeature-RNA. **(G)** The violin plots showed the value and AUC of pMT, Cell Stemness AUC, nCount-RNA, G2/M.Score, S.Score and nFeature-RNA in different cell types. **(H)** The Ro/e score was utilized to assess each cell type preference in different cell cycle phases and different groups. **(I)** The bubble graph showed KEGG enrichment analysis across different cell cycle phases. **(J)** The bubble graph showed KEGG enrichment analysis in different groups. **(K)** The box plots illustrated the distribution of each sample across the eleven cell types. **(L)** Visualization of enrichment analysis in different cell cycle phases. **(M)** Visualization of enrichment analysis in different groups.

We performed an analysis to reveal the single-cell landscape of the cervical cancer microenvironment, as shown in **Figure 1** from GSE208653, cells from nine samples (NO-HPV, N-HPV, HSIL, and CA patients) were classified into forty clusters. The distribution of cells by type, stage, and group was displayed. Using UMAP plots for dimensionality reduction, eleven cell types were identified: T cell and NK cells, endothelial cells (ECs), fibroblasts, smooth muscle cells (SMCs), epithelial cells (EPCs), B cells, plasma cells, mast cells, neutrophils, proliferating cells, and myeloid cells (**Figures 2A, B**). **Figures 2C–E** showed cell distribution in different phases (G1, G2/M, S) and groups.

We analyzed cell distribution and density for pMT, Cell Stemness AUC, nCount-RNA, G2/M.Score, S.Score, and nFeature-RNA (**Figure 2F**). **Figure 2G** illustrated the value and AUC of various cell types. We then assessed the proportions of cell types across groups and their distribution. Fibroblasts and EPCs were predominant in HSIL and the G1 phase (**Figures 2H–J**). To better understand HSIL tissue and the role of EPCs and fibroblasts in cervical cancer, we conducted functional enrichment analysis. HSIL showed enrichment in cell-substrate adhesion, collagen metabolism, phenylalanine metabolism, N-glycan biosynthesis, glutathione metabolism, and arginine/proline metabolism in G1. HSIL also displayed enrichment in apoptotic signaling, mononuclear cell differentiation, RNA splicing regulation, protein response, and translation (**Figures 2K–M**). Most cervical cancers are squamous cell carcinomas, typically originating from the squamous epithelium of the cervix (65). HPV infection is the primary cause of cervical cancer and promotes the carcinogenic transformation of cervical EPCs (66). Our findings align with the role of EPCs in cervical cancer, highlighting their key involvement in the disease.

### Characterization of tumor EPC subtypes in cervical cancer

**Figure 3:**
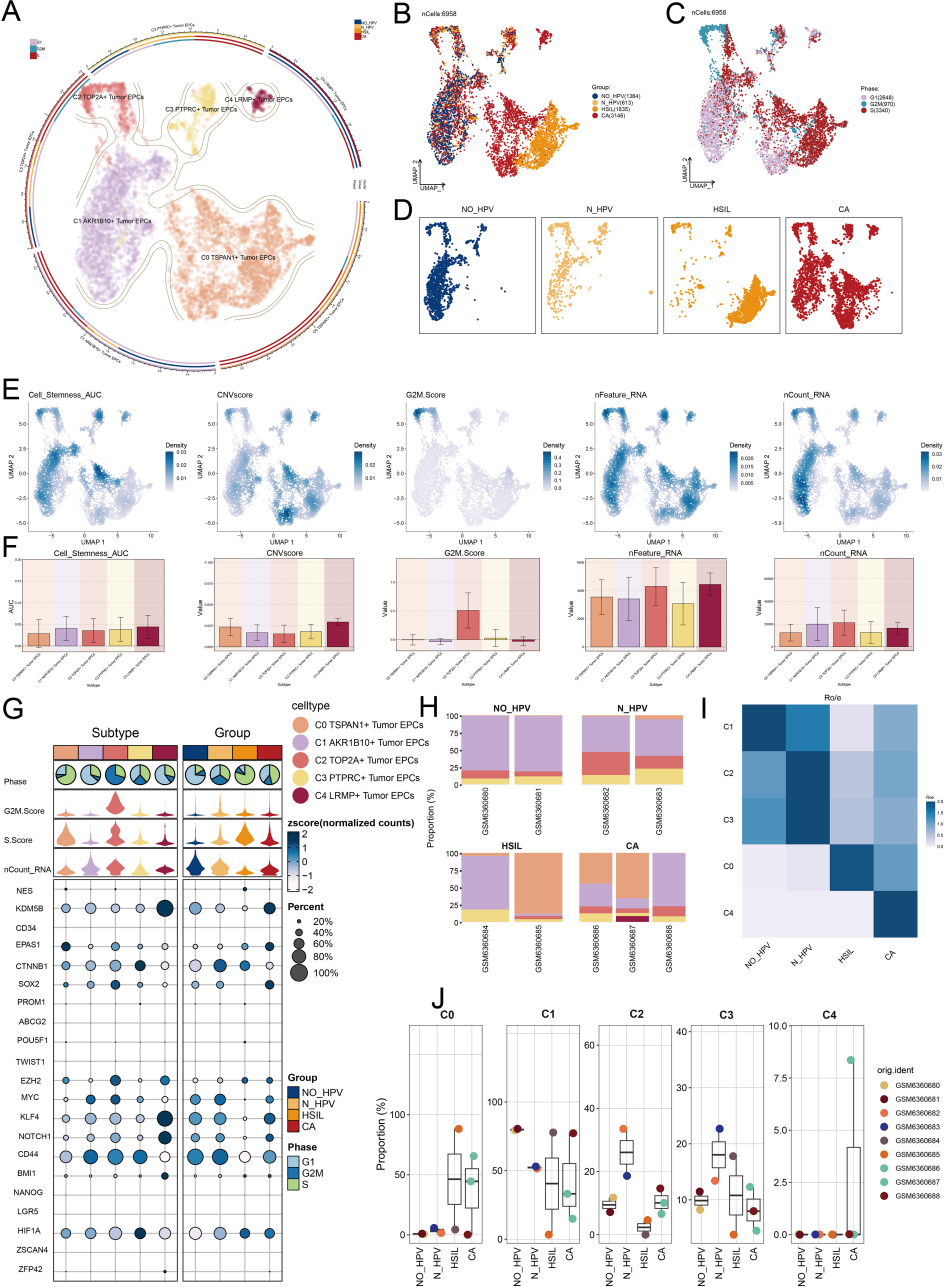
*TSPAN1+* tumor EPCs specifically expressed in HSIL and CA. **(A)** The circular plot represented the clustering of five tumor EPCs subtypes identified in cervical cancer with contour curves delineating the boundaries of each subtype. The outer axis displayed a logarithmic scale of the total number of cells within each category. **(B-D)** The UMAP plots illustrated the expression distribution of groups, phases and each group across all tumor cells. **(E)** The UMAP plots illustrated the density distribution of Cell Stemness AUC, CNV.Score, G2/M.Score, nFeature-RNA and nCount-RNA. **(F)** Bar plots illustrated the expression levels of Cell Stemness AUC, CNV.Score, G2/M.Score, nFeature-RNA and nCount-RNA across each subtype. **(G)** Bubble plots depicted the mean expression levels of the top five stemness genes in each tumor EPCs subtype. The size of each bubble corresponded to the percentage of gene expression, while the color indicated data normalization. **(H)** The stacked bar graphs displayed the distribution of each cell subtype in different sample source and group classifications. **(I)** The Ro/e score was utilized to assess the group preference of each tumor EPCs subtype. **(J)** The box plots illustrated the distribution of each subtype across different groups.

To explore tumor EPCs in the TME, we analyzed their CNVs using inferCNV with ECs as a reference (**Supplementary Figure 1**). Based on CNV levels, tumor EPCs were distinguished from ECs. We identified five tumor EPC subtypes using cell markers: C0 TSPAN1+, C1 AKR1B10+, C2 TOP2A+, C3 PTPRC+, and C4 LRMP+. Their distribution and phases were shown in **Figures 3A–D**. **Figures 3E, F** depict expression levels of Cell Stemness AUC, CNVScore, G2/M.Score, nFeature-RNA, and nCount-RNA in the subtypes. **Figure 3G** illustrated the differential expression of top five marker genes.

Our analysis showed that the C0 subtype was mainly composed of HSIL and CA cells. It had a higher proportion of these cells than other subtypes, indicating its potential role in the heterogeneity of HSIL and CA. The Ro/e preference plot confirmed a higher abundance of C0 cells in these groups (**Figures 3H–J**).

The C0 subtype had a significantly higher CNV.Score than other subtypes, aligning with the biological traits of cervical cancer. It also showed increased nFeature-RNA expression, suggesting a more malignant nature.

### Insights into metabolic and biological pathways of tumor EPC subtypes

**Figure 4:**
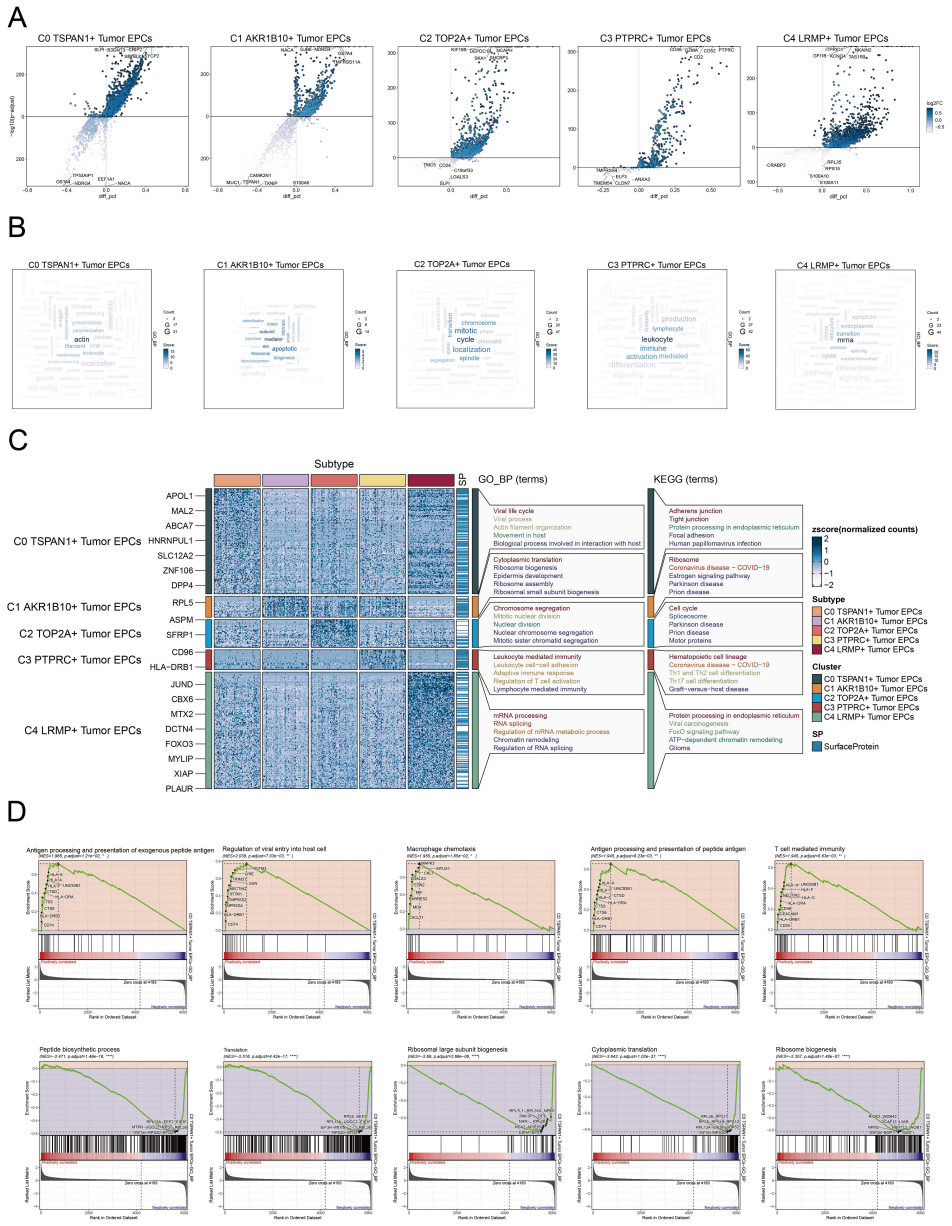
Metabolic and enrichment analysis in Tumor EPCs of cervical cancer. **(A)** The volcano plots illustrated the DEGs among the distinct cell subtypes. **(B)** The word cloud graphs depicted the biological processes linked to each tumor EPC subtype. **(C)** The heatmap displayed the top five enrichment pathways among the five clusters identified through GOBP and KEGG enrichment analysis. **(D)** GSEA analyzed ten positively or negatively enriched pathways in C0 TSPAN1+ tumor EPCs *P< 0.1**P< 0.01, ****P< 0.0001.

To investigate the biological functions of tumor EPC subtypes, we analyzed EPCs from different tissue types. We utilized a volcano plot to illustrate the differential genes in each subgroup. In the C0 subtype, significant upregulation was observed for *SLPI, B3GNT3, CRIP2, SEZ6L2*, and *SYCP2*, while genes such as *TP53AIP1, GSTA4, NDRG4, EEF1A1*, and *NACA* were downregulated (**Figure 4A**). The C0 subtype was associated with actin, filament, polymerization (**Figure 4B**). Enrichment analysis of DEGs in tumor EPC subtypes was conducted using GOBP and KEGG. The heatmap showed the top five enriched processes (**Figure 4C**). Following this, we carried out GSEA enrichment analysis for C0 subtype and identified ten biological processes that were closely related to the C0 subtype. We identified five processes with positive correlations, including antigen processing, viral entry regulation, macrophage chemotaxis, peptide antigen presentation, and T cell immunity. Conversely, five processes showed negative correlations: peptide biosynthesis, translation, ribosomal biogenesis, and cytoplasmic translation (**Figure 4D**). C0 TSPAN1+ tumor EPCs were linked to viral life cycle, actin organization, and host interactions in GOBP, and to adherens junction, tight junction, protein processing, focal adhesion, and HPV infection in KEGG.

### Pseudotime analysis to explore the developmental and differentiation traits of tumor EPC subtypes

**Figure 5:**
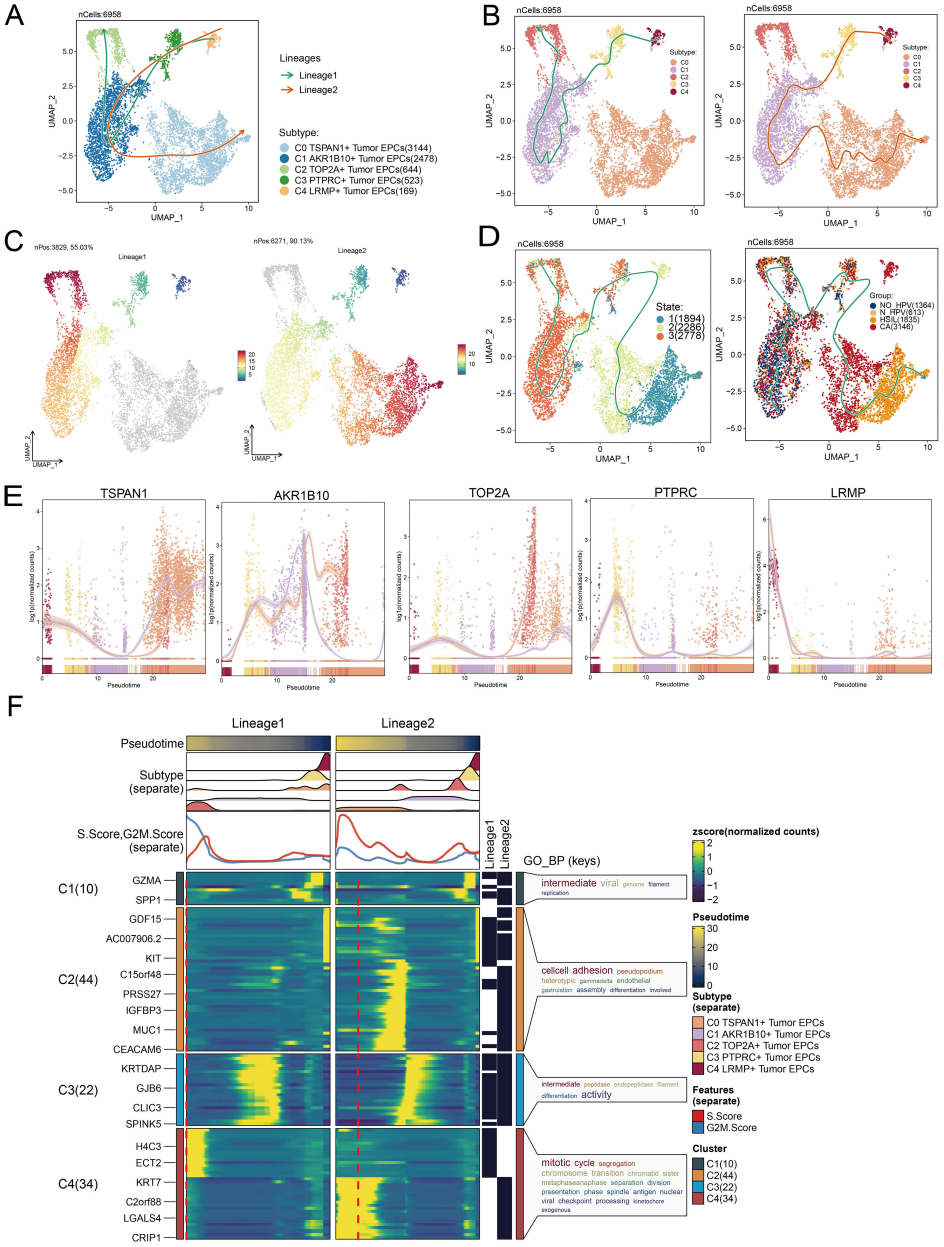
Tumor EPCs subtypes trajectory analysis. **(A-C)** The UMAP plots showed the distribution of two cell differentiation trajectories and two trajectory curves simulated by Slingshot. **(D)** The UMAP plots showed the differentiation trajectory of different state and different group by using Slingshot. The solid lines represented the differentiation trajectories, and the arrows indicated the direction of differentiation. **(E)** The dynamic trend graphs showed the expression of five marker genes. **(F)** The heatmaps showed GO enrichment pathways during the differentiation process of tumor EPCs. The top bar charts represented pseudo-time and seven different subtypes of tumor EPCs. The ridgeline plots showed the distribution density of seven subtypes across tumor EPCs, spanning various pseudo-time stages. The trajectory plots showed the expression of S.Score and G2/M. Score (red represents S.Score, blue represents G2/M. Score) as they change with pseudotime.

We analyzed the lineage and differentiation of cervical cancer tumor EPCs. **Figures 5A–C** showed that C4 LRMP+ tumor EPCs were in early differentiation stages, while C0 TSPAN1+ tumor EPCs were more differentiated. To further explore the heterogeneity of tumor EPC subtypes in cervical cancer, infer their cell lineage loci and pseudo-temporal order, and analyze the differentiation loci using Slingshot, we identified two cell lineage trajectories for tumor EPC subtypes: Lineage 1: C4 LRMP+ tumor EPCs→C3 PTPRC+ tumor EPCs→C1 *AKR1B10+* tumor EPCs→C2 TOP2A+ tumor EPCs; Lineage 2: C4 LRMP+ tumor EPCs→C3 PTPRC+ tumor EPCs→C1 *AKR1B10+* tumor EPCs→C0 TSPAN1+tumor EPCs. The C0 subtype was positioned at the end of pseudotime lineage2, a stage typically corresponding to the mature phase of cell differentiation. The runslingshot analysis of state progression trajectories further confirmed that the C0 subtype was closely related to the HSIL and CA groups. Moreover, the terminal points of the state trajectories for different groups in runslingshot were found to coincide with the C0 subtype (**Figure 5D**). Through a more detailed analysis of the trajectories of each subtype, we reaffirmed that the C0 subtype was at the far end of lineage2, displaying an obvious upward trend with data values continuously increasing over time (**Figure 5E**).

We then used GOBP enrichment analysis to examine the biological processes of the two lineages (**Figure 5F**). The dynamic timing revealed gene expression changes of tumor EPCs across the two trajectories in pseudotime.

### Cell-cell communication and MK signaling pathway visualization

**Figure 6:**
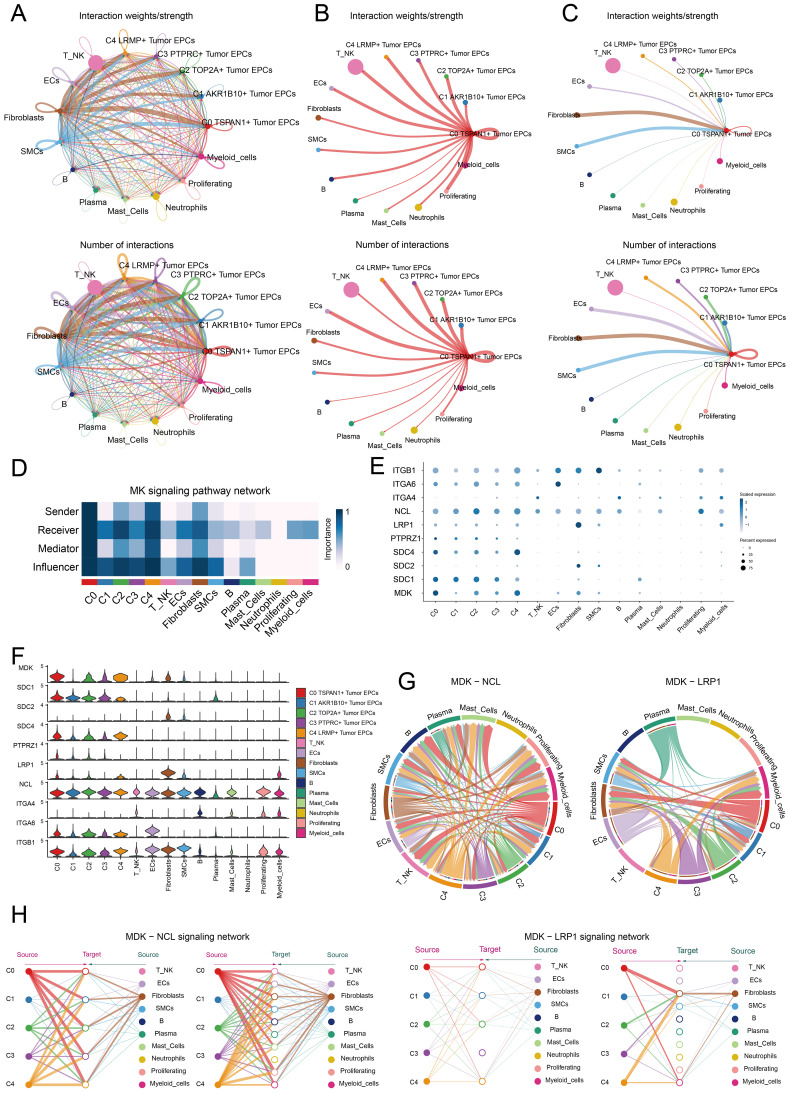
Identification of cell crosstalk networks in C0 TSPAN1+ tumor EPCs. **(A)** The circle diagrams summarized the quantity and intensity of interactions between five subtypes of tumor EPCs and ten distinct cell types, providing insights into their interconnectedness. **(B, C)** The circle diagrams showed the strength (upper) and number (lower) of interactions of C0 TSPAN1+ tumor EPCs as the source and target with other cells. **(D)** The heatmaps illustrated the roles of various proteins in different tumor EPCs types. **(E, F)** The bubble plots and violin plot illustrated the expression levels of key genes of the MK signaling pathway in different tumor EPCs types. **(G)** Chord diagrams showed the communication network of MDK-NCL and MDK-LRP1 ligand-receptor pairs. **(H)** Hierarchical graphs detailed the interactions between C0 TSPAN1+ tumor EPCs and other types of cells in MK signaling pathway.

CellChat was used to assess the communication between tumor EPC subtypes and other cell types in cervical cancer. The interactions between all cell types were summarized in terms of both number and intensity. C0 TSPAN1+ tumor EPCs showed a particularly strong effect on fibroblasts compared to other cell types. The circle graphs quantified these interactions, with C0 TSPAN1+ tumor EPCs acting as the signaling source and target (**Figures 6A–C**). The findings revealed significant communication between C0 TSPAN1+ tumor EPCs and fibroblasts.

Ligand-receptor interactions tied to the MK signaling pathway were identified. Analysis revealed that C0 TSPAN1+ tumor EPCs played multiple roles—senders, receivers, mediators, and influencers. Fibroblasts were also involved, possibly aiding the transformation to cancer-associated fibroblasts (CAFs). Fibroblasts acted as receivers, mediators, and influencers in interactions with C0 TSPAN1+ tumor EPCs. (**Figure 6D**). High communication between C0 TSPAN1+ tumor EPCs and fibroblasts was observed with the MDK-NCL and MDK-LRP1 ligand-receptor pairs (**Figures 6E, F**), and a chord diagram further validated these interactions (**Figure 6G**). C0 TSPAN1+ tumor EPCs promoted paracrine and autocrine interactions with fibroblasts, leading communication (**Figure 6H**).

The study revealed key interactions between fibroblasts and tumor EPCs in cervical cancer, suggesting that fibroblasts could transform into CAFs, promoting cancer progression.

### Exploration of TF regulatory modules

TFs regulate gene expression by attaching to specific sequences upstream of target genes, affecting cell functions.

**Figure 7:**
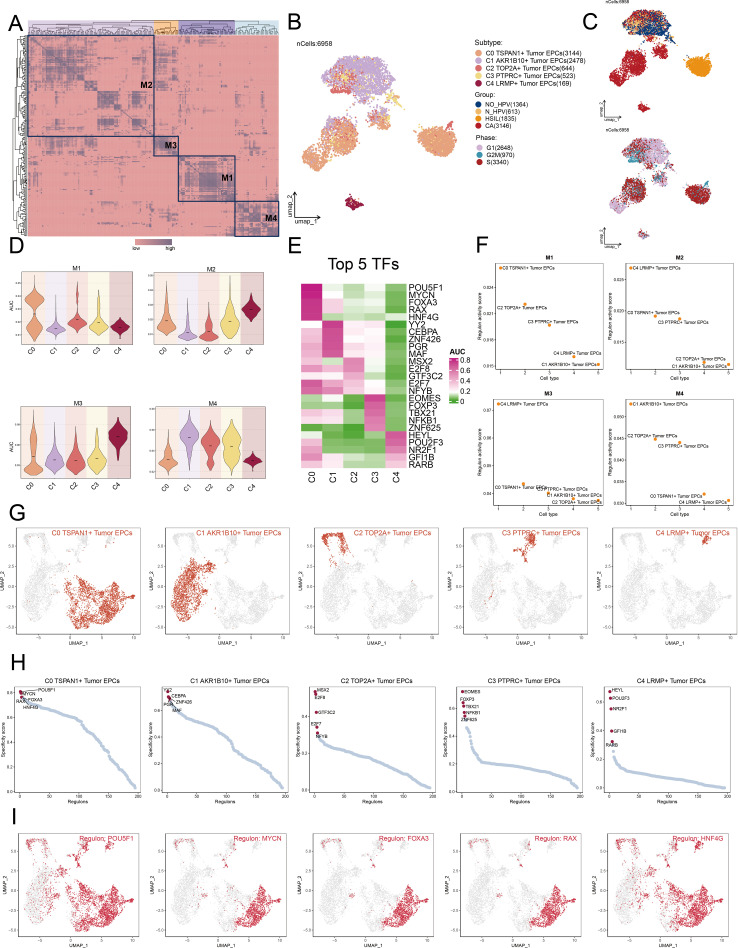
Cluster analysis of TFs and the top five TFs in each Tumor EPCs subtype. **(A)** Heatmap displayed the identification of four regulatory modules in tumor EPCs subtypes based on SCENIC regulatory rule modules and AUCell similarity scores. **(B, C)** UMAP plots colored and visualized all tumor EPCs based on the activity scores of regulatory modules, respectively, according to tumor EPCs subtypes, group and phases classifications. **(D)** The violin plots illustrated the expression levels of five tumor EPCs subtypes in four modules comprised by M1, M2, M3, M4. **(E)** The heatmap displayed top five TFs in five tumor EPCs subtypes. **(F)** The Scatter plots displayed the ranking of TF regulatory activity scores for different tumor EPCs subtypes in four modules. **(G, H)** The UMAP plots visualized the distribution of each tumor EPCs. The Scatter plots displayed the ranking of TF specificity scores for top five TFs in different tumor EPCs subtypes. **(I)** The UMAP plots visualized the distribution of top five TFs across C0 TSPAN1+ tumor EPCs.

We classified cells into four regulatory modules (M1, M2, M3, M4) using a connection specificity index matrix based on AUCell score similarities. Next, we applied the SCENIC method to cluster cervical cancer tumor EPCs by subtype, group, and phase (**Figures 7A–C**). By analyzing TF expression and regulatory activity in different tumor cell subtypes, we determined that the M1 module mainly regulated the C0 TSPAN1+ tumor EPCs (**Figure 7D**).

We reviewed the top five TFs across tumor EPC subtypes, paying particular attention to their specificity scores in different tissues (**Figures 7E, F**). Notably, C0 subtypes exhibited significant expression in M1 module. Furthermore, we studied the distribution of the subtypes, ranked the TF for each subtype (**Figures 7G, H**). We also visualized the distribution of five key regulatory factors (POU5F1, MYCN, FOXA3, RAX, and HNF4G) across C0 subtypes (**Figure 7I**).

### Establishing and evaluating the correlation in a risk prediction model

**Figure 8:**
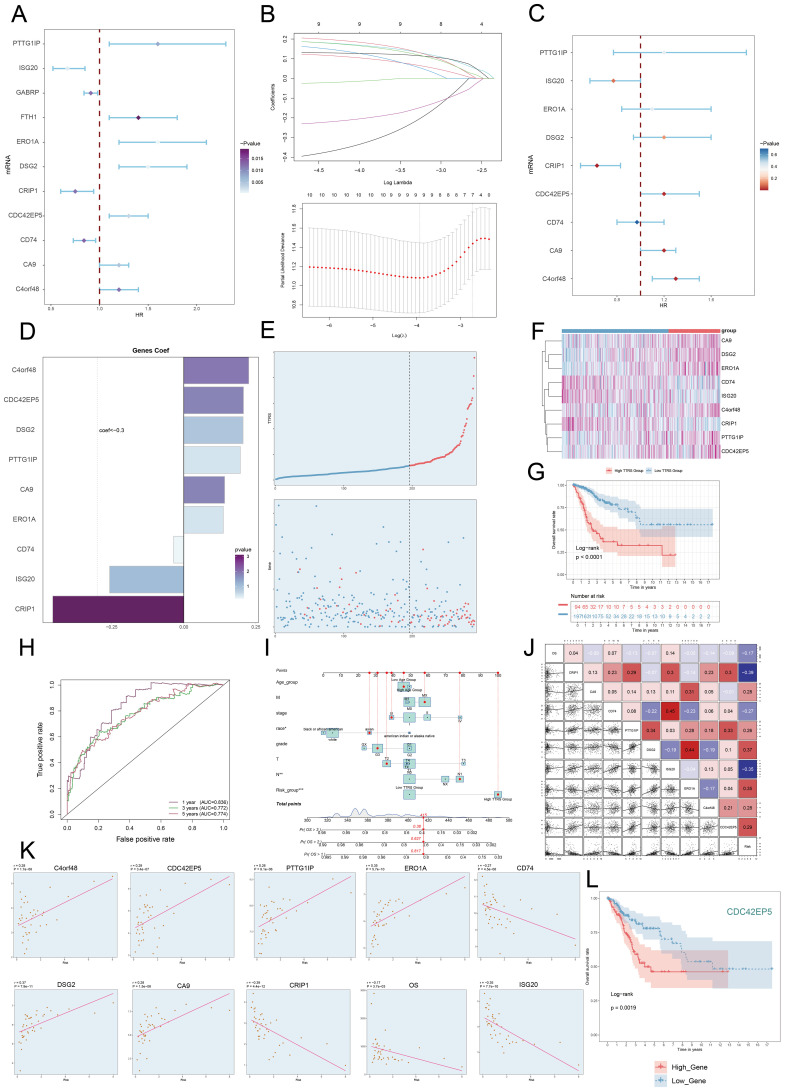
Construction and validation of the C0 TSPAN1+ Tumor EPCs risk score (TTRS) model. **(A)** Forest plot of univariate Cox regression analysis showed genes with significant differences (HR<1: protective factors, HR>1: risk factors). **(B)** LASSO regression analysis identified eleven prognostic-related genes. Each line represents the coefficient of a specific screened to have significant prognostic potential(up). The optimal parameter was determined through cross-validation (upper plot), and the LASSO coefficient curve was determined using the optimal lambda (lower plot). **(C)** Forest plot displayed nine genes obtained from multivariate Cox analysis that were associated with prognosis. **(D)** Bar graph showed the coefficient values of the genes used for model construction. **(E)** Curve chart illustrated the risk scores of high and low TTRS groups, and scatter plot depicted survival/death events over time for both groups. **(F)** The heatmap showed the expression of nine risk genes in the high TTRS group and the low TTRS group, with color scale based on normalized data. **(G)** Kaplan-Meier survival curve illustrated the survival differences among high TTRS group and low TTRS group. **(H)** Calculated the area AUC for predicting outcomes at the 1st, 3rd, and 5th years in the queue. **(I)** Nomogram showed the prediction of 1st, 2nd, and 3rd years of OS based on race, tumor clinical stage (T, M, and N), age, and risk score, with the most significant difference in the risk score group. *P< 0.1 **P< 0.01, ***P< 0.001. **(J)** Heatmap and Scatter plots demonstrated the correlation between prognostic genes, OS, and genes used in model establishment. The scatter plot showed that risk score was inversely proportional to OS. **(K)** The scatter plots showed the correlation of nine genes with OS. **(L)** Kaplan-Meier survival curve illustrated the survival differences among high *CDC42EP5* group and low *CDC42EP5* group.

Univariate Cox regression analysis was performed to evaluate each gene’s effect on prognosis (**Figure 8A**). To mitigate multicollinearity, we used IHC regression to select the key genes (**Figure 8B**). We conducted multivariate Cox regression analysis and found that *C4orf48*, *CDC42EP5*, *DSG2*, *PTTG1IP*, *CA9*, and *ERO1A* were independent unfavorable prognostic factors (HR > 1). The coefficients for these genes were calculated to measure their survival association (**Figures 8C, D**).

We calculated the *TSPAN1+* tumor EPCs score for each patient using the regression coefficients and expression levels of nine genes, as shown in the following formula: *TSPAN1+* tumor EPCs score = (0.225314727) × (*C4orf48* expression) + (0.207132307) × (*CDC42EP5* expression) + (0.205498094) × (*DSG2* expression) + (0.196215053) × (*PTTG1IP* expression) + (0.141686413) × (*CA9* expression) + (0.13721923) × (*ERO1A* expression) -(0.257186496824633)×(*ISG20* expression) - (0.454610734136572) × (*CRIP1* expression) - (-0.0352359066640519) × (*CD74* expression).

DEGs analysis was performed to examine differences between scoring groups. Based on the optimal *TSPAN1+* tumor EPCs score cut-off, TCGA participants were divided into high and low TTRS (*TSPAN1+* tumor EPCs risk score) groups. The analysis revealed that high TTRS was associated with poor clinical outcomes. Graph and scatter plot showed differences in risk scores, survival, and prognosis, with the high TTRS group having worse outcomes (**Figure 8E**). The heatmap highlighted gene expression differences between the high and low TTRS groups (**Figure 8F**). Kaplan-Meier survival curves showed worse prognosis for the high TTRS group (**Figure 8G**).

The ROC curve demonstrated the model’s ability to predict outcomes, showing AUC values at 1, 3, and 5 years (**Figure 8H**). Subgroup analysis of OS predictions across factors like age, tumor stage, and risk score revealed notable differences (**Figure 8I**). The risk score was negatively correlated with survival (**Figure 8J**), supporting the conclusion that higher risk scores resulted in shorter survival. Subsequently, we performed scatter plot analysis to assess the risk of prognostic genes and evaluated their survival curves and expression profiles in both the high and low TTRS risk groups (**Figure 8K**, **Supplementary Figures 2A, B**). The survival analysis highlighted *CDC42EP5* as the comparatively relevant gene for the high TTRS group, leading to its selection for experimental study (**Figure 8L**).

### Experimental validation *in vitro*

**Figure 9:**
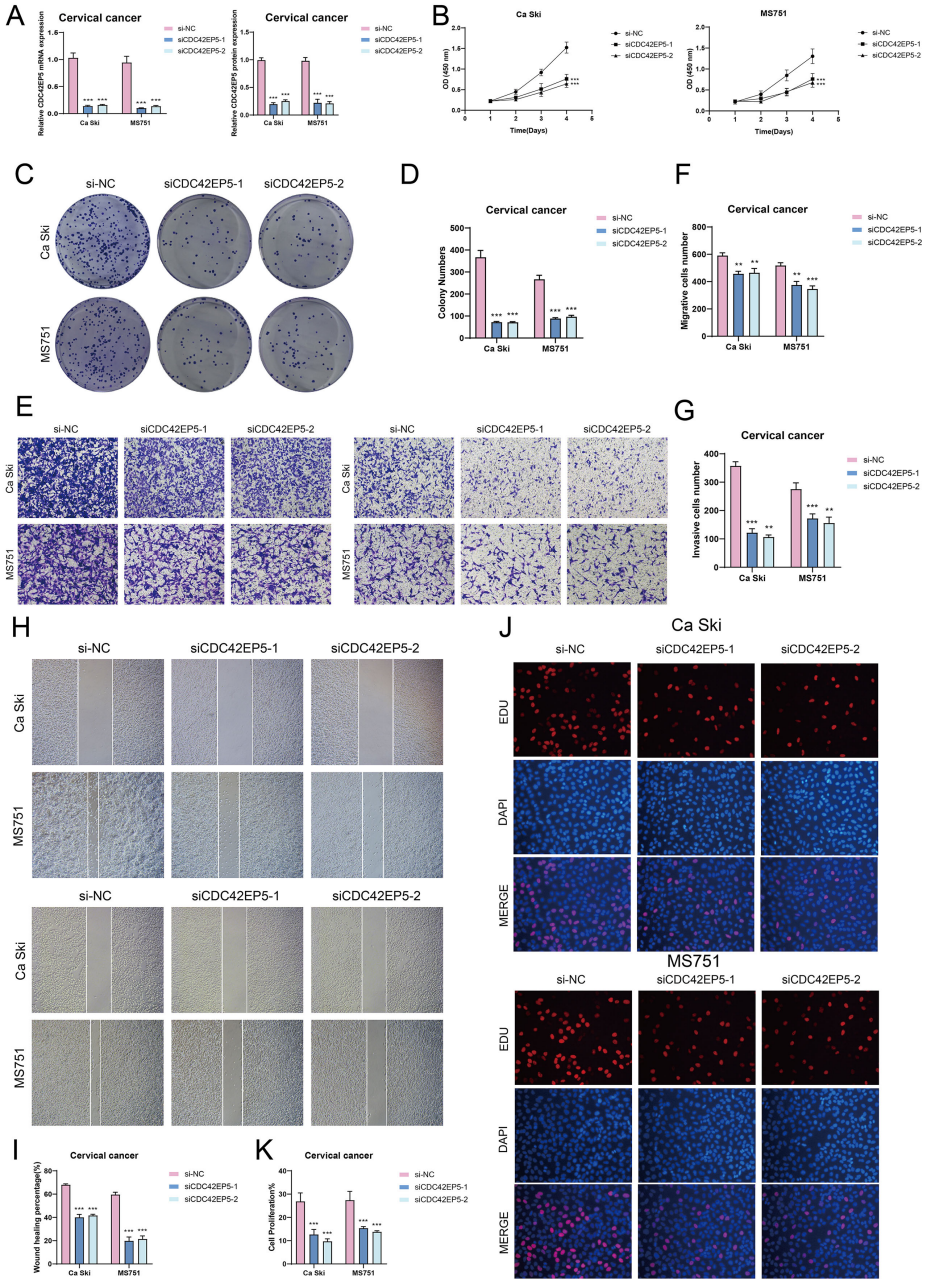
*In vitro* experiments confirmed the effects of *CDC42EP5* knockdown. **(A)** The bar graphs showed the expression of gene mRNA (left) and gene-encoded proteins (right) in the three groups of si-NC, siCDC42EP5-1, and siCDC42EP5-2 in Ca Ski and MS751 cell lines. Following *CDC42EP5* knockdown, both mRNA and protein expression levels were significantly reduced. **(B)** The CCK-8 assay results showed a notable reduction in cell viability in the Ca Ski and MS751 cell lines following the knockdown of *CDC42EP5*. **(C, D)** Colony formation assays demonstrated a significant decrease in colony numbers after *CDC42EP5* knockdown. The bar graphs showed the colony numbers in two cell lines. **(E-G)** Transwell assays showed that *CDC42EP5* knockdown suppressed the migration and invasion abilities of tumor EPCs in Ca Ski and MS751 cell lines. **(H, I)** The cell wound healing assays evaluated the migration ability of C0 TSPAN1+ tumor EPCs after treatment. Bar graph displayed a significant decrease in wound healing capabilities after *CDC42EP5* knockdown. **(J, K)** The EDU staining assay confirmed that *CDC42EP5* knockdown exerted an inhibitory effect on cell proliferation. **P< 0.01, and ***P< 0.001.

To investigate *CDC42EP5*’s role in cervical cancer, we conducted *in vitro* experiments on Ca Ski and MS751 cells. *CDC42EP5* knockdown significantly reduced both mRNA and protein levels (**Figure 9A**). This reduction led to decreased cell viability (**Figure 9B**), fewer colonies (**Figures 9C, D**), and impaired migration and invasion (**Figures 9E–I**). Additionally, Transwell and EDU assays confirmed that *CDC42EP5* knockdown inhibited cell proliferation (**Figures 9E, J, K**).

In conclusion, knockdown of *CDC42EP5* inhibited tumor cell activity, migration, invasion, and proliferation, resulting in suppressed tumor growth. This inhibition was correlated with tumor progression and adverse prognosis.

### Immunoinfiltration, investigation of DEG, functional enrichment, and drug sensitivity evaluation

**Figure 10:**
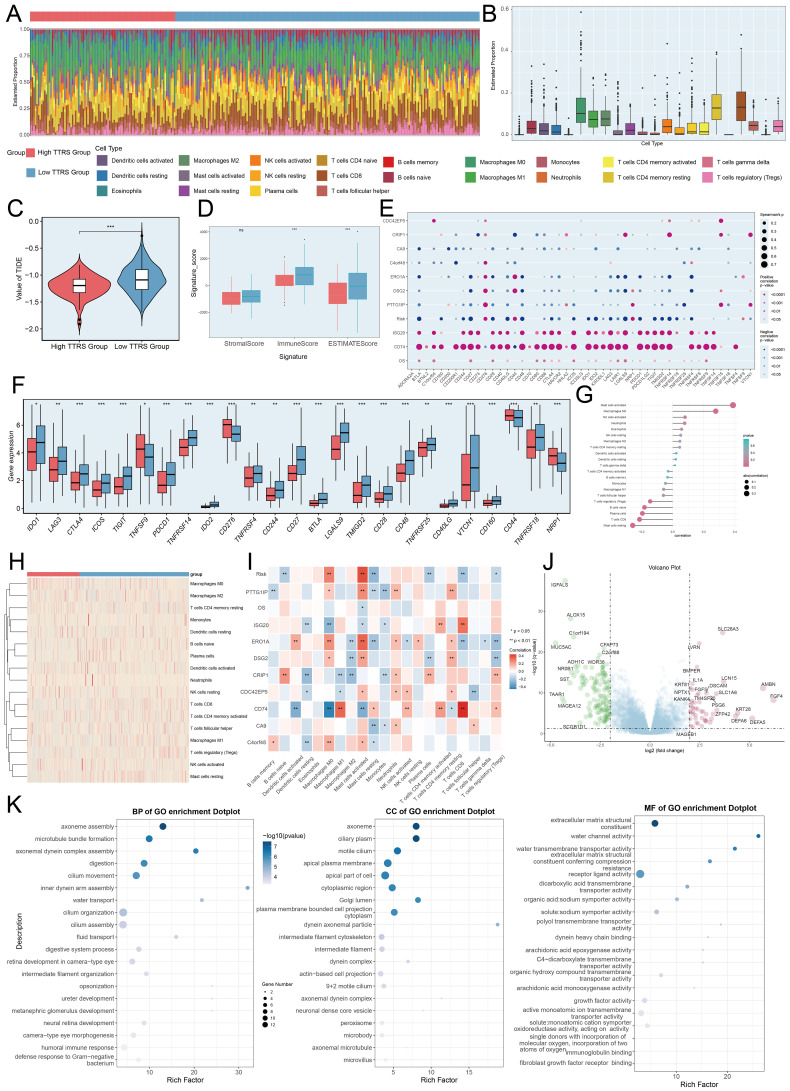
Immunoinfiltration differences, enrichment analysis across different risk groups. **(A, B)** The stacked bar graph and box plot displayed the estimated proportions of 22 types of immune cells among different risk score groups. **(C)** The violin plot illustrated TIDE expression levels in different risk score groups. **(D)** The analysis compared the differences in Stromal Score, Immune Score, and ESTIMATE Score between the high TTRS group and low TTRS group. **(E)** The bubble plots illustrated correlations among modeled genes, risk scores, OS, and immune checkpoint-related genes. **(F)** The box plot presented the expression levels of immune checkpoint-related genes in both the high TTRS group and low TTRS group. **(G, H)** The lollipop chart and heatmap demonstrated the relationship between genes and immune patterns and expression in the high TTRS group versus the low TTRS group. **(I)** The heatmap demonstrated the relationship between genes and immune patterns. **(J)** The volcano plot showed the significantly upregulated and downregulated genes in the high TTRS group and low TTRS group. **(K)** The bubble plots sequentially displayed Biological Process (BP), Cellular Component (CC), and Molecular Function (MF) categories from the GO enrichment analysis. *P< 0.05, **P< 0.01, and ***P< 0.001, “ns” was used to say that there was no significant difference.

Visualization and enrichment analysis were employed to assess gene expression and biological processes in high and low-risk groups. We began with a stacked bar chart to show cell proportions in these groups (**Figures 10A, B**). The incidence of TIDE was slightly lower in the high TTRS group (**Figure 10C**), which may have indicated to have a higher likelihood of benefiting from immune checkpoint inhibitor treatment. A low TIDE score likely reflected a better outcome from immunotherapy (67). Reduced Stromal, Immune, and ESTIMATE Scores pointed to lower cell infiltration, implying higher tumor purity and enhanced invasiveness (**Figure 10D**).

**Figure 11:**
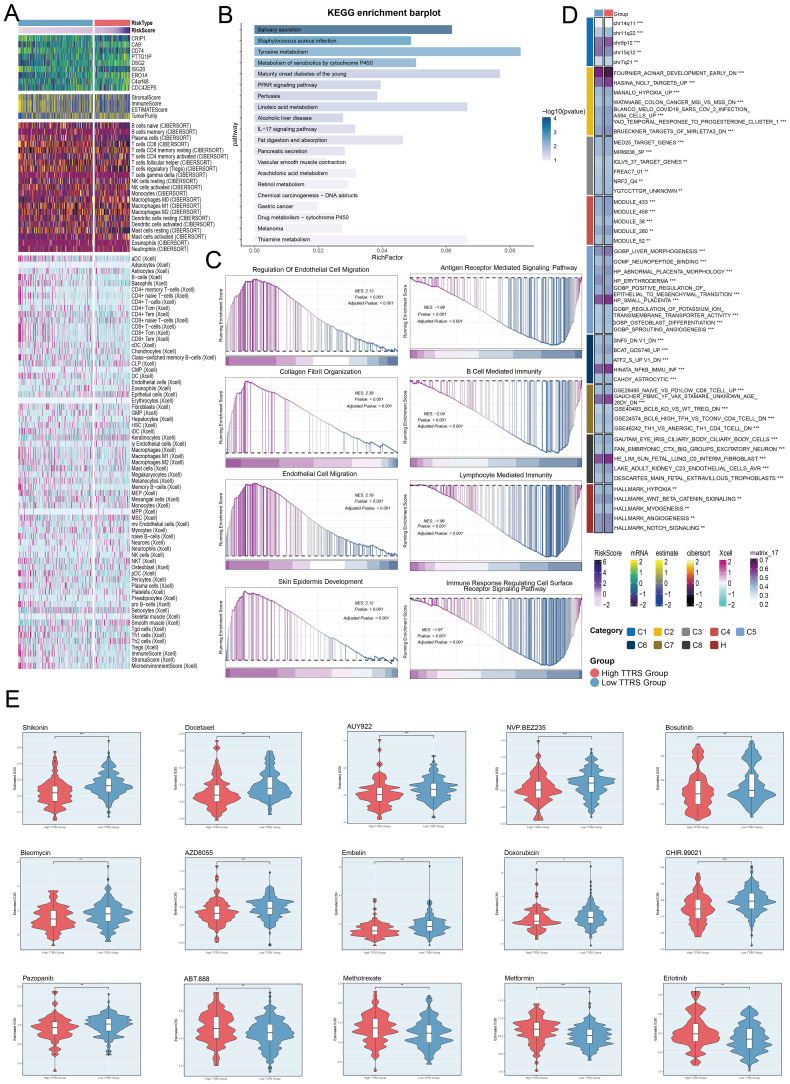
Immunoinfiltration differences, enrichment analysis, and drug sensitivity analysis across different risk groups. **(A)** The heatmap highlighted the differences in model gene expression, stromal score, immune score, ESTIMATE Score, tumor purity, and levels of immune cell infiltration calculated using CIBERSORT and Xcell between the high and low TTRS groups. Color scales were based on standardized data. **(B)** KEGG enrichment bar plot showed top 20 enrichment pathways. **(C)** Eight GSEA pathways that were positively and negatively enriched. **(D)** Detailed results of the GSVA enrichment analyses for differential gene sets between the high TTRS group and low TTRS group were presented. **(E)** The violin plots illustrated the differences in IC50 values of various chemotherapy drugs between the high TTRS group and the low TTRS group. *P< 0.05, **P< 0.01, and ***P< 0.001.

A positive correlation between *CD74* and most immune checkpoint genes was observed, while *CA9* showed a negative correlation (**Figures 10E, F**). The prognostic model revealed a positive link to M0 macrophages and a negative one to M1 macrophages, both associated with immune evasion and cancer progression. This pattern suggested that immune evasion may be contributing to poor prognosis. By analyzing the immune microenvironment, we gained a deeper understanding of macrophage polarization, TME interactions, and disease progression. We also investigated the relationship between these genes and immune checkpoint-associated genes (**Figures 10G–I**, **11A**). Volcano plots depicted DEG upregulation and downregulation (**Figure 10J**).

GO enrichment analysis revealed important biological processes, components, and molecular functions. Genes in GOBP were mostly enriched in axoneme assembly and microtubule bundle formation. For GOCC, the enrichment was primarily in the axoneme and ciliary plasm. In GOMF, the genes showed significant enrichment in extracellular matrix structural constituents and water channel activity (**Figure 10K**). Further analysis of the immune environment and KEGG pathway enrichment of differential genes revealed pathways like salivary secretion, staphylococcus aureus infection, and tyrosine metabolism. We performed a GSEA of the enriched pathways, finding positive enrichment in endothelial cell migration, collagen fibril organization, and skin epidermis development. Conversely, antigen receptor signaling, B cell immunity, lymphocyte-mediated immunity, and immune response-regulating receptor pathways showed negative trends (**Figures 11B, C**). GSVA analysis of both TTRS groups revealed functional distinctions (**Figure 11D**).

Finally, the high TTRS group displayed increased sensitivity to Shikonin, Bleomycin, and Docetaxel. Meanwhile, the low-risk group had lower IC50 values for Methotrexate, Metformin, and Erlotinib, implying these drugs could be more effective for them (**Figure 11E**).

## Discussion

TME in cervical cancer has been established as a crucial factor affecting its development, progression, and therapeutic response (68). In recent years, with the advancement of multi-omics techniques, considerable progress has been made in the study of the TME of cervical cancer, unveiling its complex cellular composition and the interactions among different cell types. The cervical cancer TME comprises various immune cells, such as tumor-infiltrating lymphocytes (TILs), regulatory T cells (Tregs), dendritic cells (DCs), and tumor-associated macrophages (TAMs). These cells, through the secretion of cytokines and their interactions, create a complex immune microenvironment that influences the recurrence and metastasis of cervical cancer (69). Research has used multi-omics analysis to highlight the tumor cell expression heterogeneity and the immune microenvironment diversity in cervical squamous cell carcinoma (66). We also employed single-cell sequencing to characterize the cervical cancer TME and found that EPCs had elevated pMT, Cell Stemness AUC, nCount-RNA, G2/M.score, S.score, and nFeature-RNA, signifying higher metabolic activity, greater transcriptional complexity, and stronger stemness. EPCs were found to be in a proliferative state, with an active cell cycle, thus holding significant research potential (70). Other studies have shown that EPCs migrated to tumor sites and differentiated into endothelial cells, promoting tumor angiogenesis, a crucial process for tumor growth and metastasis. EPCs might interact with immune cells within the TME, playing a role in the formation of an immune-suppressive environment that aids tumor cells in evading immune surveillance and accelerates tumor progression.

We further investigated and analyzed the EPCs subtypes, categorizing them into C0 TSPAN1+ Tumor EPCs, C1 AKR1B10+ Tumor EPCs, C2 TOP2A+ Tumor EPCs, C3 PTPRC+ Tumor EPCs, and C4 LRMP+ Tumor EPCs. Among these, *TSPAN1* plays a critical role in various cancers and is associated with processes such as tumor proliferation, invasion, apoptosis, autophagy, angiogenesis, stemness, and metastasis (71). *TSPAN1* can activate PI3K/AKT and EGFR/MAPK/ERK signaling pathways, enhancing tumor cell proliferation (72). *TSPAN1* may promote tumor angiogenesis by influencing EPCs in the TME. Tumor EPCs, as precursor cells involved in new blood vessel formation, play a pivotal role in tumor growth and metastasis. *TSPAN1* might affect tumor invasion and metastasis by modulating the expression of matrix metalloproteinases, which involves EPCs’ function (73). The expression of *TSPAN1* varies in different cancer types, making it a potential biomarker for cancer diagnosis and prognosis (74). The combined evaluation of *TSPAN1* with other molecules such as PTEN may improve predictions of tumor invasiveness (75). *AKR1B10*, an NAD(P)H-dependent enzyme in the aldo-keto reductase family, plays a crucial role in the proliferation and metastasis of various malignancies, including hepatocellular carcinoma (76). *AKR1B10* influences liver cancer cell proliferation and apoptosis by regulating the glycolytic pathway. It also plays a significant role in the tumor immune microenvironment, correlating positively with the infiltration levels of immune cells like macrophages and activated dendritic cells. *AKR1B10* is also significantly associated with immune regulatory factors, including a positive correlation with the immune checkpoint molecule CTLA-4 (77). *TOP2A*, a DNA topoisomerase, plays a key role in DNA replication, transcription, and chromosome structure maintenance. *TOP2A* is closely related to tumor development, invasion, therapy, and prognosis through its involvement in the cell cycle and apoptosis. *TOP2A* expression increases in various tumors, particularly non-small cell lung cancer, where its expression correlates with tumor differentiation, TNM staging, and lymph node metastasis (78). *TOP2A* interacts with Wnt3a to activate the Wnt signaling pathway, promoting tumor formation, progression, and metastasis (79). *TOP2A* is an important target for cancer drug development, with inhibitors such as doxorubicin and epirubicin being used in chemotherapy (80, 81). Co-amplification of HER2 and *TOP2A* serves as a sensitive indicator for selecting anthracycline-based chemotherapy for invasive breast cancer patients, who can benefit significantly from these treatments (82). Finally, *PTPRC*, also known as CD45, is a transmembrane glycoprotein expressed on almost all hematopoietic cells, except for mature red blood cells. *PTPRC* is a critical regulator in the activation of T and B cell antigen receptors. Disruption of *PTPRC* balance can lead to immune deficiencies, autoimmune diseases, or malignancies (83). Additionally, lymphoid-restricted membrane protein (LRMP), associated with the endoplasmic reticulum, has been found to be expressed in a developmentally regulated manner in B and T cell lines (84). Under endoplasmic reticulum stress conditions, Rictor phosphorylation at S1235 interferes with the binding between AKT and mTORC2, consequently inhibiting the phosphorylation of AKT at Ser473 via glycogen synthase kinase-3β (GSK-3β) (85).

The elevated CNV.Score observed in the C0 subtype revealed considerable genomic alterations, which not only enhanced the cellular heterogeneity but also implied a reduced stemness and increased malignant potential. This group of cells was positioned at the terminal end of the pseudotemporal lineage2 trajectory, typically indicative of the final stage in a cell’s developmental path. However, the terminal position of the C0 subtype did not signify complete differentiation but rather a state of low differentiation, which was closely associated with the tumor cells’ high proliferative and invasive capabilities (86). In pseudotemporal analysis, cells located at the end of the trajectory usually represented the most mature phase in their differentiation process, having completed multiple differentiation steps from stem or progenitor cells. Studying the cells at this terminal point allowed researchers to gain a deeper understanding of pivotal events in the differentiation process, such as the determination of cell fate, the activation or suppression of essential genes, and how cells responded to the surrounding microenvironment (87). In disease research, analyzing the terminal points in pseudotemporal differentiation proved crucial for identifying abnormal differentiation pathways in the disease state. By examining the C0 subtype, we gained insights into the key junctures in tumor cell differentiation, revealing how these critical points influenced tumor progression and patient prognosis, and potentially offering novel therapeutic targets.

*TSPAN1* likely influenced tumor progression within the TME by regulating the interaction between tumor endothelial progenitor cells and fibroblasts. CAFs constituted one of the major components of this microenvironment. Through direct contact with tumor endothelial cells and intercellular signaling, CAFs actively participated in the formation of tumor microvasculature, tumor growth, and invasion processes (88). Specifically, *TSPAN1* might have modulated intercellular signaling pathways, thereby affecting the function of both CAFs and tumor endothelial progenitor cells. This modulation could influence the dynamic balance within the TME, which plays a critical role in tumor progression.

*TSPAN1* expression in breast cancer was associated with increased migration and invasion. Knockdown of *TSPAN1* reduced these traits in MDA-MB-231 and SUM159PT cells and modified epithelial-mesenchymal transition (EMT)-related proteins expression (89). This suggests that *TSPAN1* may regulate key processes that promote metastasis in breast cancer.

Similarly, in gastric cancer, *TSPAN1* appeared to regulate CDH11 expression, which, in turn, mediated the interaction between gastric cancer cells and CAFs. The expression of CDH11 correlated closely with CAF markers, highlighting the potential role of *TSPAN1* in influencing the TME through CDH11-mediated signaling pathways. Moreover, *TSPAN1* might have activated the YAP signaling pathway, promoting the nuclear translocation of YAP and subsequently upregulating Tenascin-C (TNC) expression. This activation could enhance gastric cancer cell migration and facilitate tumor spheroid formation, further contributing to cancer progression.

Additionally, *TSPAN1* was involved in the regulation of extracellular matrix (ECM) proteins, particularly interacting with collagen I in fibroblasts. This interaction likely influenced the fibroblasts’ collagen contraction ability, which is essential for tumor cell invasion and metastasis (90). By modulating the ECM composition and structure, *TSPAN1* could directly affect the function of tumor endothelial progenitor cells, thereby influencing tumor angiogenesis and contributing to the overall progression of the tumor.

The C0 subtype, with MDK-LRP1 and MDK-NCL as its sources, both being part of the MK pathway, suggested that MDK, a heparin-binding growth factor, could interact with its receptor, LRP1. LRP1, expressed by tumor-infiltrating macrophages, promoted the differentiation of immunosuppressive macrophages (91). The expression of LRP1 in cervical cancer tissues was found to differ from that in normal cervical tissue. In cervical cancer, LRP1 expression was potentially linked to tumor development. Studies indicated that aberrant LRP1 expression in cervical cancer might contribute to poor therapeutic outcomes. High LRP1 expression was associated with low differentiation in cervical cancer and a shorter overall survival (OS) in patients, suggesting that LRP1 could serve as an effective prognostic factor for poor clinical outcomes in cervical cancer (92). Thus, MDK-LRP1 may impact cervical cancer progression and prognosis by modulating macrophage behavior, immune responses, and directly influencing cancer cell proliferation and invasion. Research using scRNA-seq and spatial transcriptomics highlighted MDK-NCL-dependent immune suppression in endometrial cancer. It found that cancer cells transmit malignant phenotypes to endothelial cells via MDK-NCL signaling, which is linked to immune suppression, suggesting a role in TME modulation. Downregulating NCL was found to inhibit tumor growth in cervical cancer through the PI3K/AKT pathway, indicating NCL’s significant role in cancer progression and its potential as a therapeutic target (93). As a growth factor, MDK contributes significantly to cancer progression and holds potential as a therapeutic target. Its expression in the TME could influence chemotherapy sensitivity, and MDK inhibitors are currently under preclinical development. Additionally, targeting MDK has been shown to eliminate IFN-γ-induced metastasis in various cancer types, further highlighting its potential role in cancer progression and metastasis (94, 95). In conclusion, the MDK-NCL signaling network may influence cervical cancer by impacting the immune microenvironment and promoting the malignant phenotype of tumor cells, with NCL expression linked to immune suppression, while MDK remains a potential target for cervical cancer therapy.

We discovered the five most prominent TFs in the C0 subgroup during our study. FOXA3, part of the FOXA family, was essential for the development and functional maintenance of organs such as the liver and pancreas (96). Similarly, HNF4G, a TF in the HNF4 family, was involved in the development and functional integrity of the liver, kidneys, and other organs (97). Moreover, MYCN, a TF from the MYC family, regulated key processes such as the cell cycle, proliferation, and apoptosis (98). As a member of the POU domain family, the POU5F1 protein regulated differentiation by binding specific octamer DNA motifs (99). RAX, another key TF, was essential for the development of the retina and brain and the differentiation of retinal ganglion cells (100).

Creation of a prognostic model that is founded on the classification of tumor cell subtypes represents a highly individualized and advanced method for forecasting the potential outcomes of patients with cancer (101). By meticulously analyzing the distinct characteristics and biological behaviors of different tumor cell subtypes, this model can provide highly tailored predictions regarding disease progression, survival rates, and potential complications. Moreover, it serves as a crucial tool for guiding personalized treatment decisions. Through our analysis of prognostic genes, we discovered that *CDC42EP5* played a vital role in prognosis. As a protein-coding gene, *CDC42EP5* belonged to the Borg family of CDC42 effector proteins. CDC42, a small GTPase, controlled the formation of F-actin structures by interacting with its downstream effector proteins (102). Research suggested that *CDC42EP5* was involved in multiple types of cancer. Knocking out *CDC42EP5* in prostate cancer increased the invasive and metastatic abilities of the cells (103). These findings indicated that *CDC42EP5* might regulate tumor invasion and metastasis, with a similar mechanism potentially present in cervical cancer. By performing further cell experiments, we provided additional evidence supporting this conclusion.

Immune checkpoints were key in regulating immune responses, with tumor cells often avoiding immune detection by increasing their expression, thereby inhibiting local immune reactions (104). Given the plentiful immune cells in cervical cancer TME, we studied variations in immune cell infiltration across distinct risk groups. Among high TTRS group, a decreased TIDE score reflected weaker immune evasion by the tumor, meaning that the immune system could more effectively detect and attack malignant cells (105, 106). As a result, these patients tended to exhibit a better response to immune checkpoint inhibitor treatment.

The immune microenvironment analysis showed that the high-risk group had lower stromal and immune scores, indicating reduced immune cell infiltration in the tumor. This reduced immune presence suggests increased tumor aggression and metastatic potential, supporting the idea that tumor purity influences invasiveness (107).

We also observed that the expression of *CD74*, a key immune checkpoint-related gene, was positively correlated with several other immune checkpoints, while *CA9* was negatively correlated. The findings suggest that immune checkpoints might be co-regulated, contributing to tumor immune evasion. Macrophage polarization was notably associated with prognosis: high levels of M0 macrophages in the high-risk group were tied to a worse prognosis, while M1 macrophages were inversely related to cancer progression (108). These findings suggest that macrophage polarization within the TME is critical for immune evasion and disease outcome.

Cervical cancer exhibited significant tumor heterogeneity, making precision medicine crucial. The study found that patients in the high TTRS group were more sensitive to drugs such as Shikonin, Docetaxel, and Camptothecin, whereas those in the low TTRS group responded better to Methotrexate, Metformin, and Erlotinib.

Shikonin inhibited the proliferation, migration, and invasion of cervical cancer cells, including HeLa and CaSki cells. It also induced apoptosis and regulated key signaling pathways, such as ROS/MAPK, Wnt/β-catenin, and FAK (109, 110). Patients in the high TTRS group exhibited greater sensitivity to Shikonin, as observed in previous studies. Docetaxel, a taxane chemotherapy, inhibits cell division by blocking microtubule depolymerization. It was used in neoadjuvant chemotherapy, combination chemotherapy, and second-line treatments for cervical cancer, demonstrating efficacy, although not without adverse effects (111). Furthermore, they enhanced therapeutic efficacy when combined with radiotherapy or chemotherapy (112–114). AUY922 was a potent HSP90 inhibitor that showed promising anticancer activity in various tumors. Since HSP90 supported the survival and proliferation of cancer cells, AUY922 disrupted this process by degrading key oncogenic proteins, ultimately leading to cell cycle arrest and apoptosis (115). NVP-BEZ235 (Dactolisib) acted as a dual PI3K/mTOR inhibitor and was shown to inhibit cervical cancer cell proliferation while inducing apoptosis in cellular assays. Furthermore, its effectiveness was enhanced in combination treatments and it exhibited radiosensitizing properties (116).

Primarily used as a tyrosine kinase inhibitor in chronic myelogenous leukemia (CML) treatment, Bosutinib also exerted tumor-suppressive effects in cervical cancer. It achieved this by downregulating Src/NF-kappaB/Survivin expression in HeLa cells (117, 118). As an antitumor antibiotic, Bleomycin was commonly used in conjunction with cisplatin for interventional and neoadjuvant chemotherapy in the treatment of locally advanced cervical cancer. Although it yielded significant results, its administration required caution due to the potential for pulmonary toxicity and other adverse effects (119). The selective mTOR kinase inhibitor AZD8055 displayed antitumor properties by inhibiting the mTORC1 and mTORC2 complexes (120). Additionally, AZD8055 enhanced the antitumor efficacy of other agents, such as HDAC inhibitors and MEK inhibitors (121). As a naturally occurring compound with antitumor properties, Embelin affected cervical cancer by targeting the SLC16A1/3 pathway. Through this action, it regulated the glycolysis and redox homeostasis of tumor cells, which ultimately suppressed the growth of cervical cancer cells (122).

In particular, doxorubicin, known for its broad-spectrum antitumor properties, was effectively applied in the treatment of cervical cancer. Nevertheless, the drug necessitated careful monitoring due to risks of cardiotoxicity and additional side effects (123). It is noteworthy that CHIR-99021, a GSK-3α/β inhibitor, was mainly applied in the basic research of cervical cancer and organoid model development. At that time, it had not entered clinical trials, and its safety and efficacy in cervical cancer treatment remained under further investigation (124).

Furthermore, Pazopanib, an oral multi-target tyrosine kinase inhibitor, had not yet become a standard treatment for cervical cancer but showed promise in improving survival and treatment outcomes when combined with chemotherapy or radiotherapy. Therefore, further clinical trials were essential to evaluate its safety and effectiveness. These drugs demonstrated distinct operational mechanisms and potential efficacy in the approach to cervical cancer. They exhibited better drug sensitivity in the high TTRS group, although some of these drugs were still in the research phase. Therefore, more clinical trials were needed in the future to verify their safety and efficacy.

Recent research mainly focused on specific cell types and signaling pathways in the cervical cancer TME, such as EPCs and their associated pathways (e.g., *TSPAN1, AKR1B10*), while studies on other cell types and molecular mechanisms were relatively scarce. This limitation might have prevented a holistic understanding of the cervical cancer TME. Additionally, the sample size was limited, and although single-cell sequencing provided a high-resolution cellular landscape, its restricted sample availability, potentially impacting the generalizability of findings. Additionally, we aimed to integrate advanced technologies such as spatial transcriptomics and single-cell multi-omics to further dissect the spatial and cellular heterogeneity of the tumor microenvironment. Experimental validation was also insufficient, as many conclusions were drawn primarily from bioinformatics analyses and *in vitro* experiments, lacking *in vivo* confirmation. Some drugs demonstrated promising effects *in vitro*, but whether they would be equally effective *in vivo* remained uncertain. Moreover, clinical applications had not yet been fully validated, as many therapeutic targets and drugs (e.g., MDK, NCL, *CDC42EP5*) were still in the research phase, with their safety and efficacy in clinical settings yet to be established.

To overcome these limitations, future research could expand the scope by investigating additional cell types and molecular mechanisms within the cervical cancer TME, such as CAFs and TANs, as well as their interactions with tumor and immune cells, to better capture the complexity of the TME. Increasing the sample size through multicenter collaborations could enhance the reliability and generalizability of research findings. Strengthening *in vivo* validation by conducting animal model experiments could help confirm the efficacy of drugs and therapeutic targets, ensuring their successful translation into clinical practice. Accelerating clinical trials for promising therapeutic targets and drugs would facilitate their validation in patients, ultimately improving treatment options for cervical cancer. Furthermore, integrating multi-omics approaches, such as genomics, transcriptomics, proteomics, and metabolomics, could provide deeper insights into the cervical cancer TME and uncover novel therapeutic targets and biomarkers. Longitudinal studies could be conducted to observe changes in the TME over disease progression and assess treatment impacts, contributing to personalized medicine. Lastly, developing individualized treatment strategies based on patient-specific characteristics and TME heterogeneity could improve therapeutic efficacy while reducing adverse effects.

## Conclusion

This study utilized multi-omics approaches to reveal the essential function of *TSPAN1* EPCs in the cervical cancer TME. However, further improvements, such as expanding the dataset, enhancing experimental validation, and optimizing clinical translation strategies, were needed to increase the credibility of the findings. Future studies could focus on developing *TSPAN1*-targeted combination therapies and applying spatial multi-omics technologies to explore the dynamic evolution of the TME, paving the way for more precise therapeutic strategies.

## Data Availability

The original contributions presented in the study are included in the article/**Supplementary Material**. Further inquiries can be directed to the corresponding author.

## Supplementary material

Supplementary Figure 1 The analysis of inferCNV. **(A, B)** The analysis of inferCNV. Using scRNA-seq data of ECs to predict CNV. Red indicated amplification, while green indicated deletion.

Supplementary Figure 2 The analysis and expression levels of prognostic genes. **(A, B)** Kaplan-Meier survival curves depicted the prognostic genes of C0 TSPAN1+ tumor EPCs and the prognostic genes expression levels across high and low TTRS groups.
