# Supplementary figures and images for: Revolutionizing cervical cancer treatment: single-cell sequencing of TSPAN1+ tumor EPCs and immune checkpoints to assess drug sensitivity and optimize therapy
Source: Front Immunol. 2025 Jul 24;16:1574174. doi: 10.3389/fimmu.2025.1574174 (PMC12328340; doi:10.3389/fimmu.2025.1574174)

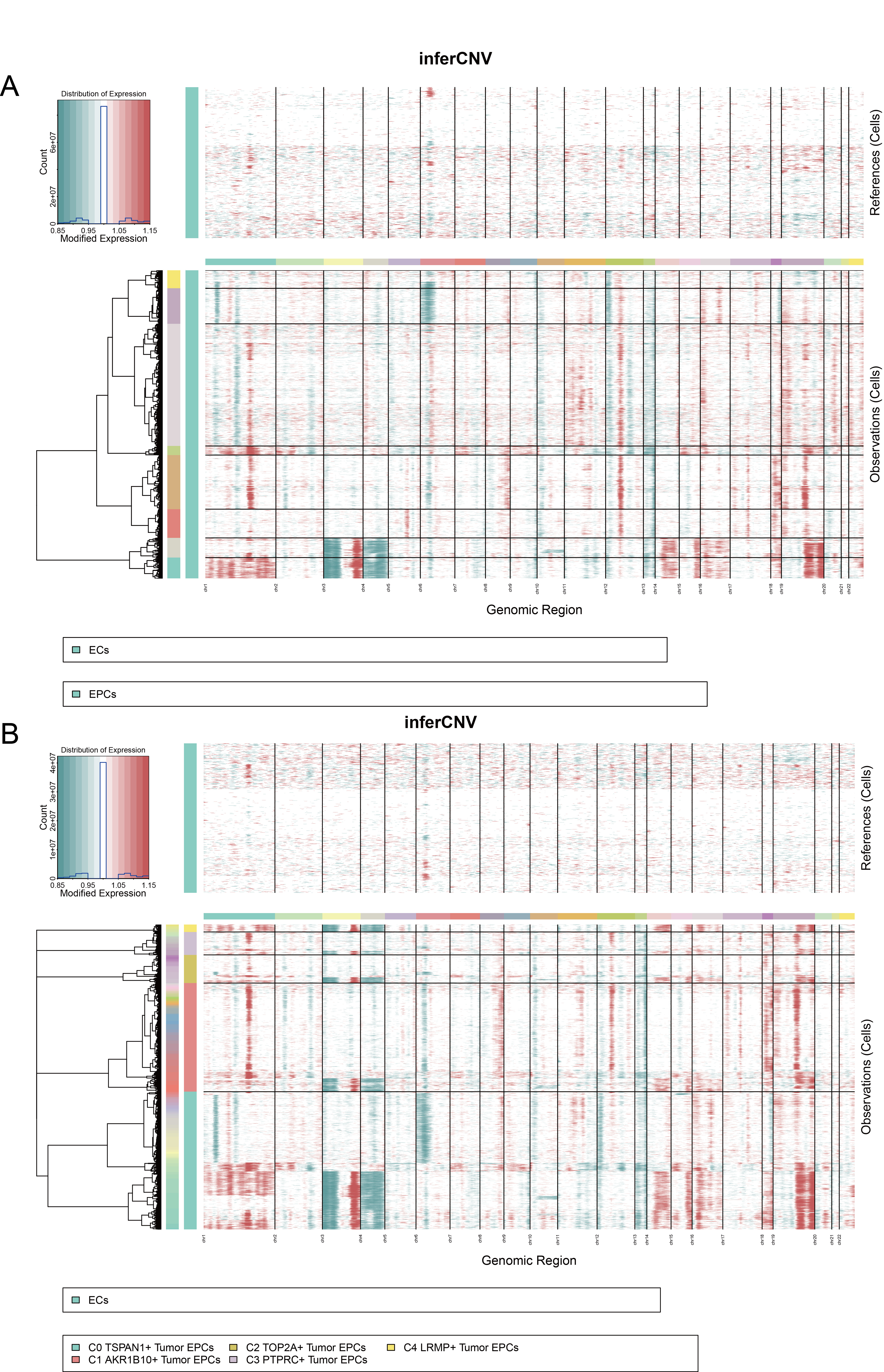

Supplement: Supplementary Figure 1 — The analysis of inferCNV. (A, B) The analysis of inferCNV. Using scRNA-seq data of ECs to predict CNV. Red indicated amplification, while green indicated deletion. [file Image1.jpeg]

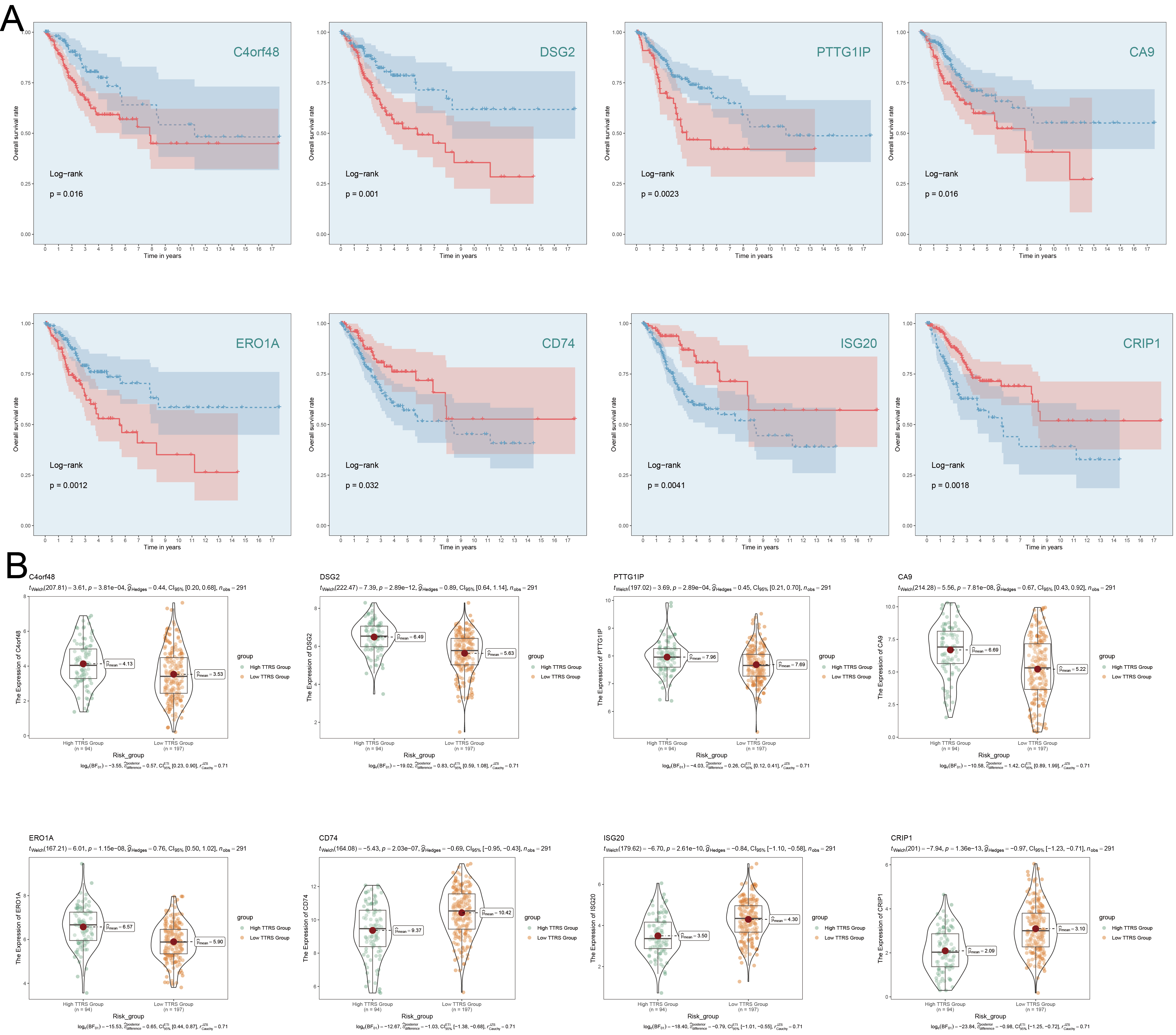

Supplement: Supplementary Figure 2 — The analysis and expression levels of prognostic genes. (A, B) Kaplan-Meier survival curves depicted the prognostic genes of C0 TSPAN1+ tumor EPCs and the prognostic genes expression levels across high and low TTRS groups. [file Image2.jpeg]
